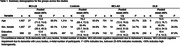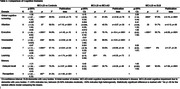# Neuropsychological test performance in mild cognitive impairment with Lewy bodies: a meta‐analysis

**DOI:** 10.1002/alz.089011

**Published:** 2025-01-03

**Authors:** Kathryn A Wyman‐Chick, Ece Bayram, Stephanie Gravett, Fabrizia D'Antonio, Joseph PM Kane, Federico Rodriguez‐Porcel, Barbara A Olson‐Bullis, Laura Bonanni, Daniel Ferreira

**Affiliations:** ^1^ HealthPartners/Park Nicollet Struthers Parkinson’s Center, Golden Valley, MN USA; ^2^ University of California San Diego, La Jolla, CA USA; ^3^ Karolinska University Hospital, Stockholm, Sodermanland Sweden; ^4^ Sapienza University of Rome, Roma, Lazio Italy; ^5^ Queen’s University Belfast, Belfast, Northern Ireland United Kingdom; ^6^ Medical University of South Carolina, Charleston, SC USA; ^7^ HealthPartners Institute, Bloomington, MN USA; ^8^ University G. d'Annunzio of Chieti‐Pescara, Chieti Italy; ^9^ Mayo Clinic, Rochester, MN USA

## Abstract

**Background:**

Prodromal dementia with Lewy bodies (DLB) research criteria were recently published and further work is needed to characterize the neuropsychological profile and refine clinical criteria. We performed a meta‐analysis to compare the cognitive performance of mild cognitive impairment with Lewy bodies (MCI‐LB) groups to controls, MCI due to Alzheimer’s disease (MCI‐AD), and DLB.

**Method:**

Following PRISMA guidelines, we searched the literature for **1)** English‐language studies on MCI‐LB; **2)** published January 1990‐March 2023, **3)** with ≥1 standardized cognitive measure; and **4)** comparison data from controls, MCI‐AD, and/or DLB. Pooled means with 95% confidence intervals were calculated for demographics. Effect sizes were calculated for group comparisons on cognitive domains. Random effects meta‐analysis models were used for group comparisons.

**Result:**

There were 3440 participants from 27 studies across 10 countries. Mean age did not differ between groups. The percentage of female subjects was lower in the MCI‐LB group than the MCI‐AD group. Mean years of education for the MCI‐LB group was lower than the controls, but similar to MCI‐AD and DLB groups (Table 1). Compared to controls, the MCI‐LB group had lower scores for the global screening, executive function, visuospatial, language, learning, and memory domains. Compared to MCI‐AD, MCI‐LB performed better on learning and delayed recall, but similarly across other domains. Compared to DLB, MCI‐LB had better scores for global screening, executive function, language, and delayed recall (Table 2). Recognition for MCI‐LB vs. MCI‐AD; visuospatial, learning, and recognition for MCI‐LB vs. DLB could not be assessed, as fewer than 3 studies reported this data.

**Conclusion:**

Cognitive measures can help differentiate MCI‐LB from controls and DLB. Neuropsychological measures of learning and memory may be required to discriminate MCI‐LB from MCI‐AD in clinical practice but global screening measure scores appear insufficient in this regard. High heterogeneity across MCI‐LB studies highlights the need for harmonized neuropsychological research protocols.